# Radiological findings of partial expression pentalogy of Cantrell and other multiple congenital anomalies: A rare case report^☆^

**DOI:** 10.1016/j.radcr.2022.05.083

**Published:** 2022-06-28

**Authors:** Ratih Sulistyowati, Anggraini Dwi Sensusiati

**Affiliations:** a Department of Radiology, Faculty of Medicine Universitas Airlangga – Dr. Soetomo General Academic Hospital, Surabaya, Indonesia; b Department of Radiology, Faculty of Medicine Universitas Airlangga – Universitas Airlangga Hospital, Surabaya, Indonesia

**Keywords:** Pentalogy of Cantrell, Congenital anomaly malformation, Ectopia cordis, Computed tomography scans, Pediatric

## Abstract

Pentalogy of Cantrell is a rare syndrome of anomalous malformation. In the present case, the syndrome was initially diagnosed as a complete pentad, including a supra-umbilical abdominal wall defect, a sternal defect, pericardial defects, an anterior diaphragmatic defect, and heart malformation. Diagnosis required several imaging modalities, including computed tomography (CT) and magnetic resonance imaging (MRI). In this case report, we present an 8-month-old female patient with a thoracic wall defect with ectopia cordis and a bilateral cleft lip and palate. In addition, a head CT scan showed craniosynostosis, hypogenesis of the corpus callosum, and tonsillar cerebellar herniation. Thoracoabdominal CT revealed herniation of the transverse colon up to the subcutaneous layer, diaphragmatic hernia, atrial septal defects (ASD), ventral septal defects (VSD), and a persistent left superior vena cava (PLSVC). A multidisciplinary approach is required for the optimal management of this syndrome. We describe a female infant who presented with pentalogy of Cantrell syndrome and include the findings from postnatal CT imaging.

## Introduction

Rare cases of Pentalogy of Cantrell have been reported since the first case was characterized by Cantrell and colleagues in 1958. It was consist of supra umbilical abdominal wall defect, sternal defect, pericardial defects, anterior diaphragmatic defect and heart malformation [1]. In the most severe cases, the heart herniates through the defect in the diaphragm, resulting in ectopia cordis. Omphalocele with ectopia cordis is the hallmark of this syndrome. The prevalence of pentalogy of Cantrell has been reported to range from 1:65,000 to 5.5:1 million live births [2].

In 1972, Toyama suggested an alternative classification system to describe the more commonly occurring variants of this syndrome: [2]

Class 1: definitive diagnosis (all 5 defects present).

Class 2: probable diagnosis (4 defects present, including ventral wall abnormalities and cardiac defects).

Class 3: incomplete expression (various combinations of defects present, including a sternal abnormality).

The pathogenesis of this syndrome is not fully understood. Occurrence of the complete pentalogy is rare, and the survival rate of patients with complete pentalogy is low, with outcomes dependent on the severity of the cardiac malformation, extracardiac defects, and other associated anomalies [3].

Cleft lip, with or without cleft palate and encephalocele, tends to be specifically associated with ventral midline anomalies within the spectrum of pentalogy of Cantrell [4].

We report a case of an incomplete pentalogy of Cantrell and highlight the importance of diagnosis and the wide range of anomalies associated with this syndrome.

*Informed consent was obtained from the patient's mother (legal guardian) for the publication of this case report.*

## Case description

The 8-month-old female patient described in the present case report was born in Pamekasan Hospital in East Java. The baby was delivered via normal vaginal birth, and the child cried spontaneously at birth. There was no history of maternal illness during the pregnancy, and the mother did not receive antenatal care. There was no history suggestive of exposure to teratogens during the pregnancy. Furthermore, there is no family history of birth defects.

**Fig. 1:**
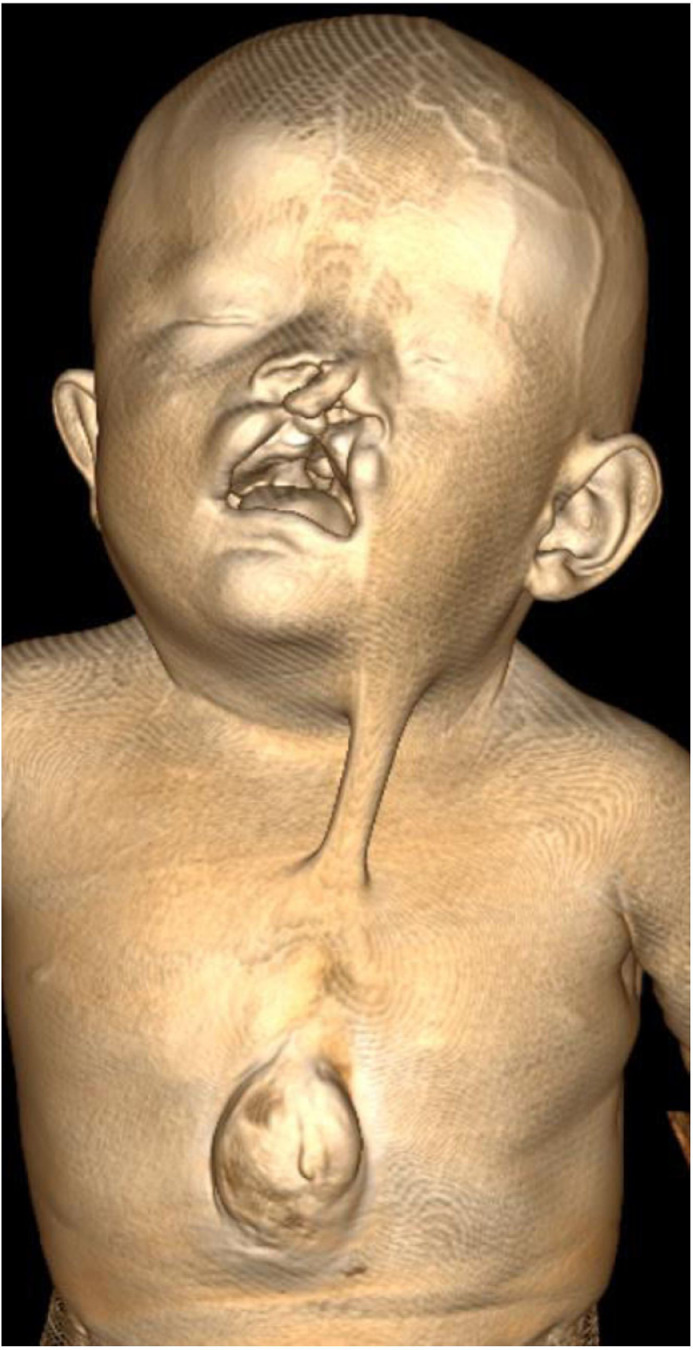
Three-dimensional (3D) volume rendering showing a defect in the thoracic wall, with ectopia cordis, and a bilateral cleft lip and palate.

Physical examination revealed a defect in the thoracic wall with ectopia cordis, and a bilateral cleft lip and palate (Fig. 1). Chest examination showed an obvious anterior chest-wall defect, which was covered by pink and membranous skin over the epigastrium. The cardiac impulse was visible. Palpation of the chest wall revealed a complete sternal defect. Breath sounds were clearly heard all over the chest area, but only the first and second heart sounds were heard over the precordium. The heart rate was 130 beats per minute. The umbilical stump was intact, with no obvious anomaly of the umbilicus.

**Fig. 2:**
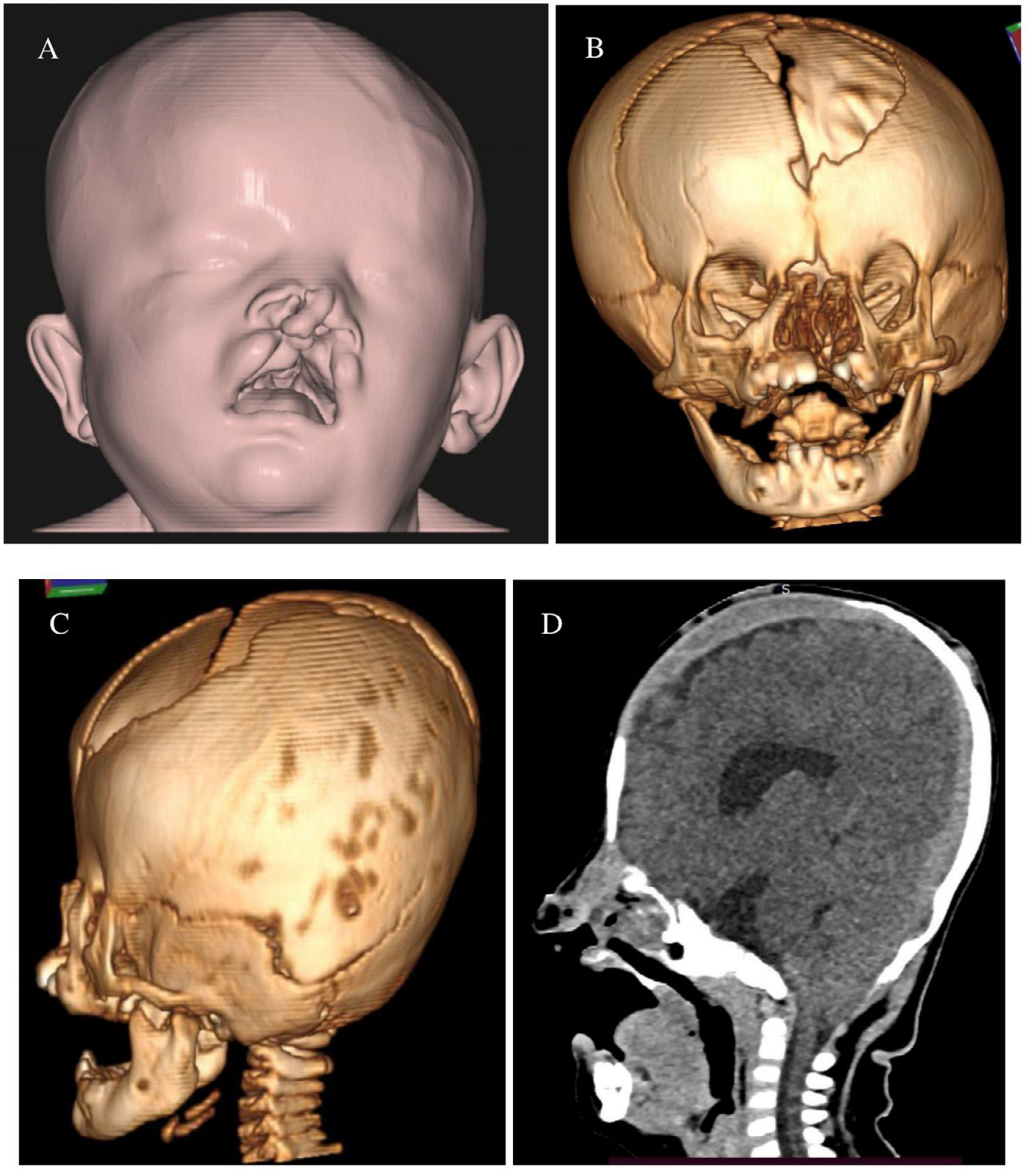
Head three-dimensional (3D) volume rendering (A) and head bone 3D reconstruction (B) showed defects in the nasal bone, right and left medial orbital wall, and cleft lip and palate. The 3D reconstruction of the left side (C) showed a craniosynostosis at the left lambdoid suture, left coronaria suture, and frontalis suture. Sagittal head computed tomography (CT) scan of the cranial window (D) revealed hypogenesis of the corpus callosum and tonsillar cerebellar herniation.

The patient subsequently underwent postnatal evaluation in our radiology department. A contrast computed tomography (CT) scan of the head and three-dimensional (3D) reconstruction showed craniosynostosis at the left lambdoid suture, left coronal suture, and frontalis suture, leading to an appearance of turricephaly, along with maxillofacial defects in the nasal bone, orbital cavity, and cleft lip and palate. With the brain window setting, we identified hypogenesis corpus callosum and tonsillar herniation (Fig. 2).

**Fig. 3:**
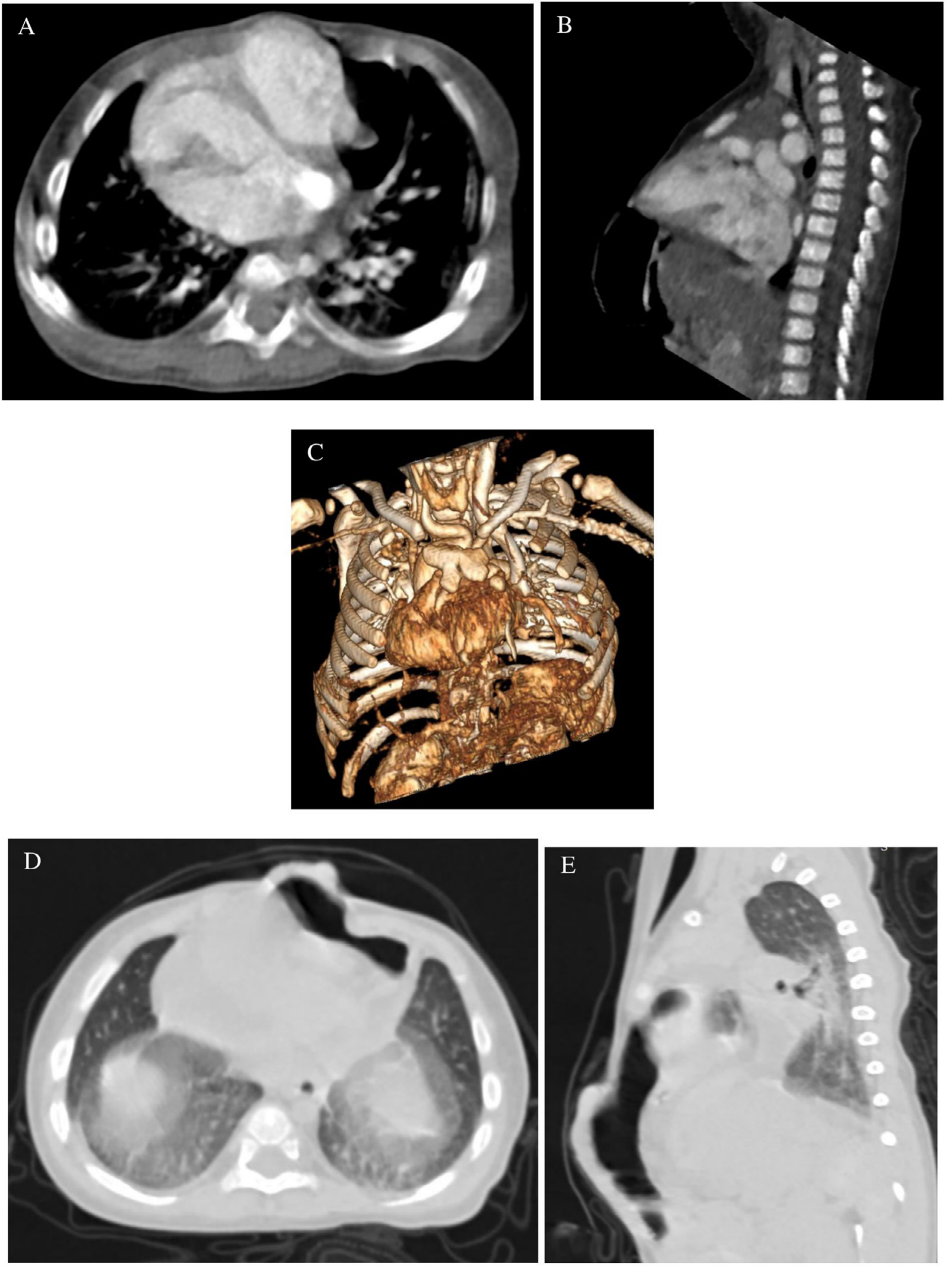
Axial (A) and sagittal (B) chest computed tomography (CT) scans showed a defect of the sternum (red arrow) and a herniated left ventricle of the heart. Bone 3-dimensional (3D) reconstruction (C) showed a defect in the sternum. Axial (D) and sagittal (E) upper abdominal CT scans revealed a defect of the anterior diaphragm, with a hernia in the transverse colon through the defect and up to the subcutaneous layer.

**Fig. 4:**
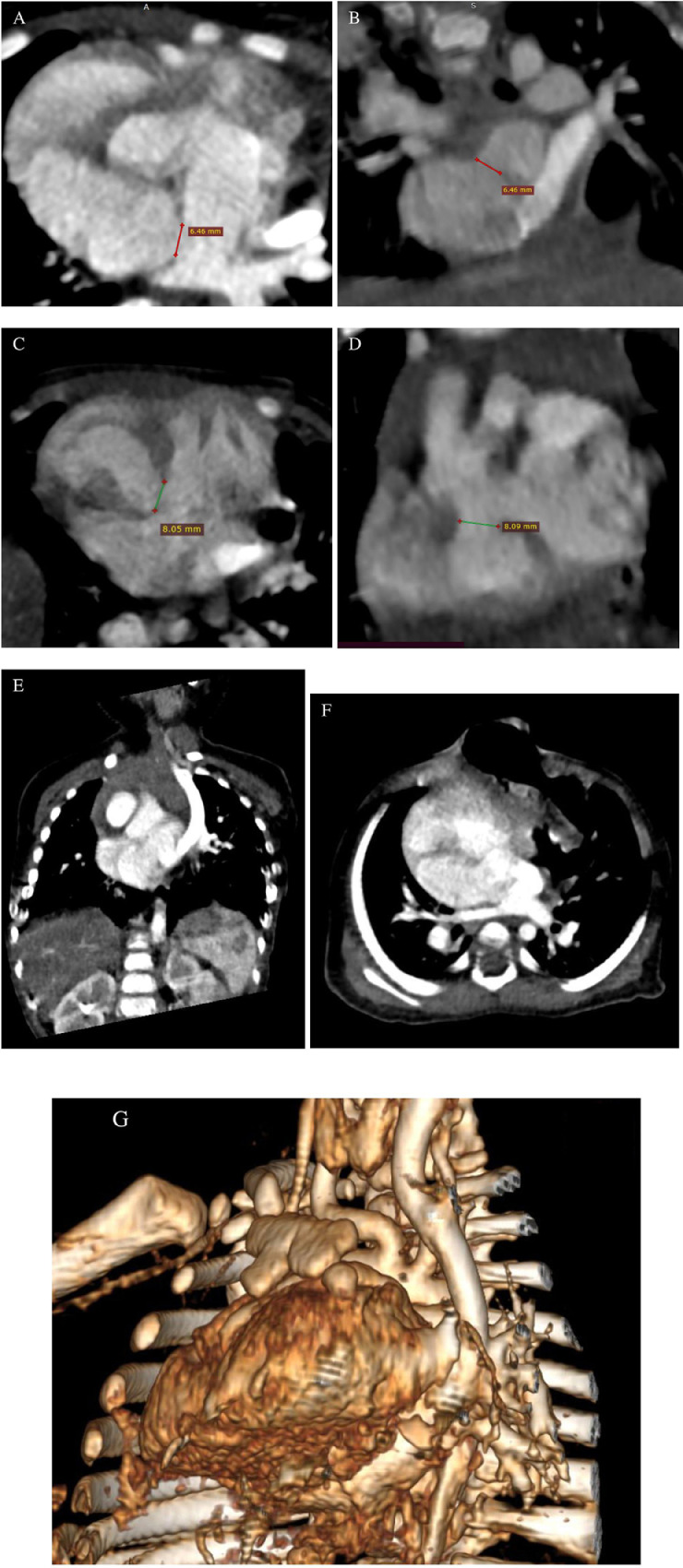
Axial (A) and coronal (B) chest computed tomography (CT) scans of the mediastinal window showed an atrial septal defect. Axial (C) and coronal (D) chest CT scans also revealed a ventricle septal defect. Coronal (E), axial (F), and 3D reconstruction (G) showed a persistent left superior vena cava (PLSVC).

The patient also underwent contrast chest and abdominal CT. The results showed a defect of the sternum accompanied by left ventricle herniation of the heart outside the thoracic cavity that was covered with a thin serous membrane. The upper abdominal wall and anterior diaphragm were absent, with a hernia in the transverse colon through the defect and up to the subcutaneous layer (Fig. 3). We also found an atrial septal defect, ventricle septal defect, and a persistent left superior vena cava (PLSVC; Fig. 4).

## Discussion

The etiology and pathogenesis for pentalogy of cantrell are not well understood and are thought to have a heterogeneous origin. Cantrell et al. suggested that a developmental failure in a segment of the lateral mesoderm occurred around embryonic days 14-18. Consequently, the transverse septum of the diaphragm does not develop, and the ventromedial migration of the paired mesodermal folds of the upper abdomen fails to occur. Due to the subsequent sternal and abdominal wall defects, the organs may eviscerate [1]. Abnormal migration of the splanchnic and somatic mesoderm, along with premature breakage of the vitelline sac, results in a midline defect. Malformation of the transverse septum of the diaphragm occurs due to the abnormal migration of myoblasts. In addition, premature atrophy of the cardinal vein leads to associated pericardial defects [1,5,6].

Most reported cases of pentalogy of Cantrell are thought to be sporadic in occurrence; however, mutations in genes located on the X chromosome have been associated with ventral midline disruptions. An X-linked dominant inheritance pattern has been observed in some families [4,7]. In such cases, the ventral midline is regarded as a developmental field, and embryologic anomalies in single genes involved in the development of this field are responsible for the wide range of midline defects [4,8,9].

A specific gene that could be responsible for this disorder has not yet been identified; however, a recent case report described a novel maternally inherited microduplication of chromosome 15q21.3 on the postnatal chromosomal microarray of a newborn with a prenatal diagnosis of pentalogy of Cantrell. This region includes the gene *ALDH1A2*, which encodes for retinaldehyde dehydrogenase type 2, an enzyme with a critical role in normal cardiac and diaphragm development, thus demonstrating a biologically plausible association with this disorder [10].

Kumar et al. proposed that variations of pentalogy of Cantrell could be classified according to the number of defects: Class 1 is defined as a definitive diagnosis in which all five defects are present; Class 2 is defined as a probable diagnosis in which 4 defects are present, including intracardiac and abdominal wall abnormalities; Class 3 is defined as an incomplete diagnosis in which at least 2 of Cantrell's anomalies are present, one of which is sternal [11].

Ghidini et al. described complete and incomplete variants of the pentalogy [6]. Therefore, it is plausible that, as more cases are recognized as pentalogy of Cantrell, we will increase our awareness of other associated anomalies and variants not previously described [12].

CT is an effective diagnostic tool for infants suspected of having pentalogy of Cantrell and generally provides high spatial resolution, truly multiplanar capabilities, and fast acquisition times without the need for anesthesia. However, the ionizing radiation and iodine contrast required for CT must be weighed against these benefits [13]. In the present case, chest and abdominal CT scans with contrast revealed defects including ectopia cordis – the hallmark of this syndrome – along with pulmonary stenosis, atrial septal defects (ASD) and ventral septal defects (VSD), which have been reported to occur in 33%, 52%, and 100% of cases, respectively [1].

Cantrell suggested that a developmental failure in the lateral mesoderm around embryonic days 14-18 leads to partial developmental failure of the transverse septum of the diaphragm, in which migration of the paired mesodermal fold does not occur [1]. Failure of the transverse septum to develop, as well as abnormal development of the myocardium, leads to diaphragmatic and cardiac defects, respectively [14]. The triple A syndrome gene mutation, which is mapped to the Xq25-q26.1 area, has resulted in cases of sternal roll infusion and multiple hearts, as well as diaphragm and anterior abdominal wall abnormalities [4,9].

Our patient also presented with a rare PLSVC vascular anomaly that began at the junction of the left subclavian and internal jugular veins and passed through the left side of the mediastinum adjacent to the aortic arch. It mostly drains into the right atrium through the coronary sinus. Although PLSVC is rare among vascular anomalies, it is the most common thoracic venous anomaly. For the most part, PLSVC is asymptomatic and is typically detected incidentally in diagnostic examinations [15].

In addition to the findings described above, the results of a head CT scan with 3-dimensional reconstruction revealed another congenital anomaly in the present case, namely craniosynostosis, which is a condition of premature fusion of one or more cranial sutures, resulting in characteristic skull deformity and facial asymmetry. Approximately 85% of cases of primary craniosynostosis occur as isolated conditions, and the remaining 15% are part of a multisystem syndrome [16].

Imaging is essential for obtaining an accurate diagnosis, planning surgical treatments, post-treatment evaluation, and identification of other anatomic abnormalities and complications associated with craniosynostosis. In this regard, because of the superior bone depiction, 3D CT reconstruction is the primary imaging technique used for craniosynostosis [15].

Treatment strategies and the prognosis of pentalogy of Cantrell depend on the severity of intra and extracardiac defects, pulmonary hypoplasia, the extent of abdominal wall defects, cerebral anomalies, and diaphragmatic herniation [17]. The mortality rate increases with complete presentation or in the presence of ectopia cordis [18].

The fusion defect leaves the mediastinal viscera unprotected, resulting in an increased risk of harm to the underlying mediastinal organs. Surgical repair should be performed in the first weeks of life to restore the protective function of the skeleton by establishing bony integrity, as soon as possible, and to take advantage of the high flexibility of the chest wall in newborns [19].

A multidisciplinary team should follow up on milder forms of this disorder in order to determine the best time for delivery. After delivery, repair of the omphalocele should be carried out as soon as possible. Repair of the sternal, diaphragmatic, and pericardial defects can also be attempted at the same time [15].

## Conclusion

Pentalogy of Cantrell is a rare anomalous malformation syndrome, the etiology and pathogenesis of which are not fully understood and are considered to be of heterogeneous origin. The diagnosis of the present case required several imaging modalities, including CT scans and magnetic resonance imaging (MRI). In general, CT scans can diagnose and categorize pentalogy of Cantrell and provide high spatial resolution, truly multiplanar capabilities, and fast acquisition times, without the need for anesthesia. Moreover, during the diagnostic process, it is important for clinicians to be aware of the range of imaging possibilities and other assessments.
